# *trans*-Carbonyl­chloridobis[tris(naph­thalen-1-yl)­phosphane-κ*P*]rhodium(I) acetone trisolvate

**DOI:** 10.1107/S1600536811038505

**Published:** 2011-09-30

**Authors:** Reinout Meijboom

**Affiliations:** a Research Centre for Synthesis and Catalysis, Department of Chemistry, University of Johannesburg, PO Box 524, Auckland Park, 2006, Johannesburg, South Africa

## Abstract

In the title compound, *trans*-[RhCl{P(C_10_H_7_)_3_}_2_(CO)]·3C_3_H_6_O, where P(C_10_H_7_)_3_ is trinaphthyl­phosphine, the Rh—P bond lengths are 2.3360 (10) and 2.3258 (10) Å, while the Rh—Cl bond length is 2.3525 (11) Å. The coordination around the Rh atom shows a slightly distorted square-planar arrangement.

## Related literature

For related compounds see: Otto (2001 ▶); Otto *et al.* (2000 ▶); Chen *et al.* (1991 ▶); Kuwabara & Bau (1994 ▶). Symmetrical square-planar complexes of Rh, Ir, Pd and Pt often crystallize with the metal atom on a crystallographic centre of symmetry, thus imposing a disordered packing arrangement. The present study is part of an ongoing investigation into determining which factors govern a disordered packing mode. The title compound is one of the few examples which does not show disorder along the carbonyl/chlorido axis. For similar non-disordered compounds, see: Burgoyne *et al.* (2010 ▶); Makhoba *et al.* (2011 ▶).
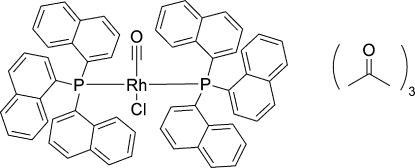


## Experimental

### 

#### Crystal data

[RhCl(C_30_H_21_P)_2_(CO)]·3C_3_H_6_O
*M* *_r_* = 1165.48
Monoclinic, 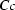

*a* = 10.1306 (15) Å
*b* = 27.673 (4) Å
*c* = 20.201 (3) Å
β = 94.610 (4)°
*V* = 5645.0 (14) Å^3^
*Z* = 4
Mo *K*α radiation
μ = 0.46 mm^−1^
*T* = 100 K
0.34 × 0.22 × 0.04 mm

#### Data collection

Bruker APEXII CCD diffractometer
Absorption correction: multi-scan (*SADABS*; Bruker; 2004 ▶) *T*_min_ = 0.888, *T*_max_ = 0.983
31682 measured reflections
13902 independent reflections
11679 reflections with *I* > 2σ(*I*)
*R*_int_ = 0.057

#### Refinement

*R*[*F* ^2^ > 2σ(*F* ^2^)] = 0.046
*wR*(*F* ^2^) = 0.098
*S* = 0.99
13902 reflections
709 parameters
2 restraints
H-atom parameters constrained
Δρ_max_ = 0.90 e Å^−3^
Δρ_min_ = −0.56 e Å^−3^
Absolute structure: Flack (1983 ▶), 6894 Friedel pairs
Flack parameter: −0.001 (19)

### 

Data collection: *APEX2* (Bruker, 2005 ▶); cell refinement: *SAINT-Plus* (Bruker, 2004 ▶); data reduction: *SAINT-Plus* and *XPREP* (Bruker, 2004 ▶); program(s) used to solve structure: *SHELXS97* (Sheldrick, 2008 ▶); program(s) used to refine structure: *SHELXL97* (Sheldrick, 2008 ▶); molecular graphics: *DIAMOND* (Brandenburg, 2005 ▶); software used to prepare material for publication: *WinGX* (Farrugia, 1999 ▶).

## Supplementary Material

Crystal structure: contains datablock(s) global, I. DOI: 10.1107/S1600536811038505/mw2021sup1.cif

Structure factors: contains datablock(s) I. DOI: 10.1107/S1600536811038505/mw2021Isup2.hkl

Additional supplementary materials:  crystallographic information; 3D view; checkCIF report

## Figures and Tables

**Fig. 1.**
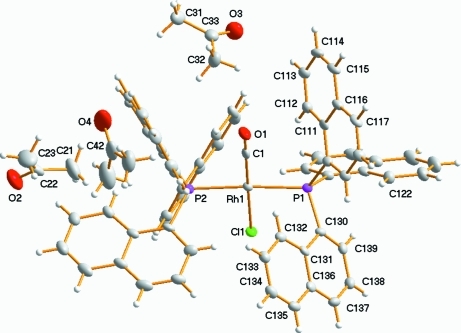
The structure of the title compound, showing 50% probability displacement ellipsoids. For the C atoms, the first digit indicates ring number and the second digit indicates the position of the atom in the ring. Some labels have been omitted for clarity, all rings have been numbered in the same, systematic manner.

## supplementary crystallographic information

### Comment

Symmetrical square-planar complexes of Rh, Ir, Pd and Pt often crystallize with the metal atom on a crystallographic centre of symmetry, thus imposing a disordered packing arrangement (Otto, 2001; Otto *et al.*, 2000; Chen *et al.*, 1991; Kuwabara & Bau, 1994). The present study is part of an ongoing investigation into determining which factors govern a disordered packing mode and reports the structure of *trans*-carbonylchloridobis(trinaphthylphosphine)rhodium(I). The compound is one of the few crystallographic examples of these complexes which does not show disorder along the carbonyl/chloro axis (Burgoyne *et al.*, 2010; Makhoba *et al.*, 2011). The coordination around the Rh atom shows a slightly distorted square-planar arrangement.

### Experimental

Tri-naphthylphosphine (0.08 g, 0.21 mmol) was dissolved in acetone (5 cm^3^). A solution of dichlorotetracarbonyldirhodium(I) (0.02 g, 0.05 mmol) in acetone (5 cm^3^) was added to the phosphine solution. The mixture was stirred for 5 minutes, after which the solution was left to crystallize. Yellow crystals of the title compound were obtained.

### Refinement

The aromatic,and methyl H atoms were placed in geometrically idealized positions (C—H = 0.95–0.98) and constrained to ride on their parent atoms with *U*_iso_(H) = 1.2*U*_eq_(C) for aromatic and *U*_iso_(H) = 1.5*U*_eq_(C) for methyl H atoms respectively. Methyl torsion angles were refined from electron density.

### Crystal data

**Table:**

[RhCl(C_30_H_21_P)_2_(CO)]·3C_3_H_6_O	*F*(000) = 2416
*M**_r_* = 1165.48	*D*_x_ = 1.371 Mg m^−^^3^
Monoclinic, *C**c*	Mo *K*α radiation, λ = 0.71073 Å
Hall symbol: C -2yc	Cell parameters from 5353 reflections
*a* = 10.1306 (15) Å	θ = 2.3–25.8°
*b* = 27.673 (4) Å	µ = 0.46 mm^−^^1^
*c* = 20.201 (3) Å	*T* = 100 K
β = 94.610 (4)°	Plate, yellow
*V* = 5645.0 (14) Å^3^	0.34 × 0.22 × 0.04 mm
*Z* = 4	

### Data collection

**Table:**

Bruker APEXII CCD diffractometer	13902 independent reflections
Radiation source: sealed tube	11679 reflections with *I* > 2σ(*I*)
graphite	*R*_int_ = 0.057
φ and ω scans	θ_max_ = 28.3°, θ_min_ = 1.5°
Absorption correction: multi-scan (*SADABS*; Bruker; 2004)	*h* = −13→13
*T*_min_ = 0.888, *T*_max_ = 0.983	*k* = −34→36
31682 measured reflections	*l* = −26→26

### Refinement

**Table:**

Refinement on *F*^2^	Secondary atom site location: difference Fourier map
Least-squares matrix: full	Hydrogen site location: inferred from neighbouring sites
*R*[*F*^2^ > 2σ(*F*^2^)] = 0.046	H-atom parameters constrained
*wR*(*F*^2^) = 0.098	*w* = 1/[σ^2^(*F*_o_^2^) + (0.0401*P*)^2^] where *P* = (*F*_o_^2^ + 2*F*_c_^2^)/3
*S* = 0.99	(Δ/σ)_max_ = 0.003
13902 reflections	Δρ_max_ = 0.90 e Å^−^^3^
709 parameters	Δρ_min_ = −0.56 e Å^−^^3^
2 restraints	Absolute structure: Flack (1983), 6894 Friedel pairs
Primary atom site location: structure-invariant direct methods	Flack parameter: −0.001 (19)

### Special details

**Table:**

Geometry. All e.s.d.'s (except the e.s.d. in the dihedral angle between two l.s. planes) are estimated using the full covariance matrix. The cell e.s.d.'s are taken into account individually in the estimation of e.s.d.'s in distances, angles and torsion angles; correlations between e.s.d.'s in cell parameters are only used when they are defined by crystal symmetry. An approximate (isotropic) treatment of cell e.s.d.'s is used for estimating e.s.d.'s involving l.s. planes.
Refinement. Refinement of *F*^2^ against ALL reflections. The weighted *R*-factor *wR* and goodness of fit *S* are based on *F*^2^, conventional *R*-factors *R* are based on *F*, with *F* set to zero for negative *F*^2^. The threshold expression of *F*^2^ > σ(*F*^2^) is used only for calculating *R*-factors(gt) *etc*. and is not relevant to the choice of reflections for refinement. *R*-factors based on *F*^2^ are statistically about twice as large as those based on *F*, and *R*- factors based on ALL data will be even larger.

### Fractional atomic coordinates and isotropic or equivalent isotropic displacement parameters (Å^2^)

**Table:**

	*x*	*y*	*z*	*U*_iso_*/*U*_eq_	
C1	0.5700 (4)	0.70768 (13)	0.71336 (19)	0.0197 (8)	
C10	0.4720 (4)	0.75940 (14)	0.90434 (19)	0.0189 (8)	
H10	0.5327	0.7468	0.9368	0.023*	
C21	0.3148 (6)	0.91871 (18)	0.8501 (3)	0.0592 (16)	
H21A	0.2548	0.8918	0.8483	0.089*	
H21B	0.394	0.9104	0.877	0.089*	
H21C	0.3369	0.9266	0.8061	0.089*	
C22	0.2511 (5)	0.96117 (15)	0.8796 (2)	0.0351 (11)	
C23	0.1343 (6)	0.9827 (2)	0.8417 (3)	0.0630 (17)	
H23A	0.1624	0.9997	0.8038	0.094*	
H23B	0.0915	1.0049	0.8696	0.094*	
H23C	0.0735	0.9576	0.8271	0.094*	
C31	0.1743 (5)	0.63458 (18)	0.9130 (2)	0.0402 (12)	
H31A	0.1715	0.6102	0.9467	0.06*	
H31B	0.0865	0.6401	0.8928	0.06*	
H31C	0.2087	0.664	0.9326	0.06*	
C32	0.2779 (5)	0.65282 (16)	0.8050 (2)	0.0368 (11)	
H32A	0.3291	0.6379	0.7726	0.055*	
H32B	0.3225	0.6814	0.822	0.055*	
H32C	0.1922	0.6614	0.7846	0.055*	
C33	0.2621 (4)	0.61795 (16)	0.8611 (2)	0.0309 (9)	
C41	0.1187 (8)	0.9664 (2)	0.6213 (3)	0.080 (2)	
H41A	0.1095	0.958	0.6667	0.12*	
H41B	0.1449	0.9996	0.6187	0.12*	
H41C	0.1848	0.9462	0.6038	0.12*	
C42	−0.0094 (7)	0.95927 (17)	0.5819 (3)	0.0521 (16)	
O4	−0.1061 (6)	0.94247 (14)	0.6066 (2)	0.0895 (17)	
C110	0.6912 (4)	0.63432 (13)	0.61001 (18)	0.0184 (7)	
C111	0.5784 (4)	0.60753 (12)	0.62852 (18)	0.0176 (7)	
C112	0.5610 (4)	0.59302 (13)	0.6952 (2)	0.0218 (9)	
H112	0.6255	0.6008	0.7289	0.026*	
C113	0.4520 (4)	0.56801 (13)	0.7101 (2)	0.0245 (8)	
H113	0.443	0.5589	0.7538	0.029*	
C114	0.3527 (4)	0.55567 (13)	0.6605 (2)	0.0247 (9)	
H114	0.2779	0.539	0.6715	0.03*	
C115	0.3664 (4)	0.56821 (13)	0.5966 (2)	0.0252 (9)	
H115	0.3007	0.5597	0.5639	0.03*	
C116	0.4795 (4)	0.59413 (13)	0.57838 (19)	0.0210 (8)	
C117	0.4953 (4)	0.60580 (13)	0.51121 (19)	0.0232 (8)	
H117	0.4315	0.5963	0.4781	0.028*	
C118	0.6040 (4)	0.63101 (14)	0.4950 (2)	0.0218 (9)	
H118	0.6135	0.6387	0.4509	0.026*	
C119	0.7015 (4)	0.64540 (13)	0.54427 (17)	0.0193 (8)	
H119	0.7744	0.6628	0.5323	0.023*	
C120	0.8843 (4)	0.60061 (13)	0.71304 (18)	0.0187 (7)	
C121	0.9266 (4)	0.55858 (13)	0.67798 (19)	0.0208 (8)	
C122	0.9226 (4)	0.55473 (14)	0.60798 (19)	0.0242 (8)	
H122	0.8889	0.5803	0.5821	0.029*	
C123	0.9668 (4)	0.51458 (14)	0.5774 (2)	0.0305 (10)	
H123	0.9624	0.5129	0.5313	0.037*	
C124	1.0194 (5)	0.47556 (15)	0.6160 (2)	0.0365 (11)	
H124	1.0524	0.4486	0.5953	0.044*	
C125	1.0220 (5)	0.47715 (15)	0.6830 (2)	0.0344 (10)	
H125	1.0537	0.4507	0.7079	0.041*	
C126	0.9767 (4)	0.51870 (14)	0.7157 (2)	0.0247 (9)	
C127	0.9821 (4)	0.52022 (14)	0.7864 (2)	0.0302 (9)	
H127	1.0148	0.4938	0.811	0.036*	
C128	0.9401 (4)	0.55975 (15)	0.8181 (2)	0.0281 (9)	
H128	0.9425	0.5602	0.8642	0.034*	
C129	0.8928 (4)	0.60008 (14)	0.78146 (19)	0.0210 (9)	
H129	0.8665	0.6273	0.804	0.025*	
C130	0.9467 (4)	0.68404 (13)	0.63052 (17)	0.0172 (7)	
C131	0.9307 (4)	0.73167 (12)	0.60455 (17)	0.0166 (7)	
C132	0.8109 (4)	0.75808 (13)	0.60300 (18)	0.0191 (8)	
H132	0.7358	0.7434	0.6175	0.023*	
C133	0.8031 (4)	0.80487 (14)	0.58056 (19)	0.0219 (8)	
H133	0.7233	0.8215	0.5805	0.026*	
C134	0.9140 (4)	0.82788 (14)	0.55780 (18)	0.0242 (8)	
H134	0.9077	0.8596	0.5429	0.029*	
C135	1.0306 (4)	0.80377 (14)	0.55755 (18)	0.0231 (8)	
H135	1.1034	0.8191	0.5417	0.028*	
C136	1.0428 (4)	0.75567 (14)	0.58109 (18)	0.0200 (9)	
C137	1.1648 (4)	0.73095 (14)	0.58231 (19)	0.0234 (8)	
H137	1.2378	0.7462	0.5664	0.028*	
C138	1.1768 (4)	0.68526 (14)	0.60638 (19)	0.0233 (8)	
H138	1.2576	0.6693	0.6064	0.028*	
C139	1.0687 (4)	0.66206 (13)	0.63110 (18)	0.0203 (8)	
H139	1.0794	0.631	0.6484	0.024*	
C210	0.5165 (4)	0.77527 (13)	0.84513 (18)	0.0178 (7)	
C211	0.4235 (4)	0.79589 (12)	0.79625 (18)	0.0184 (7)	
C213	0.4599 (4)	0.81645 (13)	0.73540 (18)	0.0214 (8)	
H213	0.5483	0.8165	0.7261	0.026*	
C214	0.3672 (4)	0.83595 (14)	0.69095 (19)	0.0260 (9)	
H214	0.3935	0.8499	0.6522	0.031*	
C215	0.2317 (4)	0.83552 (14)	0.70244 (19)	0.0283 (9)	
H215	0.1691	0.8485	0.6712	0.034*	
C216	0.1933 (4)	0.81590 (14)	0.7596 (2)	0.0262 (9)	
H216	0.1038	0.8152	0.7668	0.031*	
C217	0.2870 (4)	0.79663 (12)	0.80827 (18)	0.0195 (8)	
C218	0.2472 (4)	0.77876 (13)	0.86930 (19)	0.0229 (8)	
H218	0.158	0.7786	0.8771	0.027*	
C219	0.3391 (4)	0.76176 (13)	0.91667 (18)	0.0204 (8)	
H219	0.3126	0.7517	0.9575	0.024*	
C220	0.7764 (4)	0.74587 (13)	0.90737 (18)	0.0183 (7)	
C221	0.7635 (4)	0.69568 (14)	0.92564 (18)	0.0204 (8)	
C222	0.6681 (4)	0.66430 (14)	0.89414 (19)	0.0225 (8)	
H222	0.6079	0.6763	0.8609	0.027*	
C223	0.6626 (5)	0.61682 (15)	0.9116 (2)	0.0283 (10)	
H223	0.5984	0.5969	0.8905	0.034*	
C224	0.7539 (5)	0.59760 (15)	0.9616 (2)	0.0323 (10)	
H224	0.7519	0.5649	0.9723	0.039*	
C225	0.8447 (5)	0.62740 (15)	0.9938 (2)	0.0276 (10)	
H225	0.903	0.6149	1.0275	0.033*	
C226	0.8523 (4)	0.67687 (14)	0.97734 (18)	0.0220 (8)	
C227	0.9478 (4)	0.70750 (15)	1.01019 (19)	0.0265 (9)	
H227	1.0053	0.6952	1.0443	0.032*	
C228	0.9567 (4)	0.75456 (15)	0.99269 (19)	0.0244 (9)	
H228	1.0188	0.7745	1.0155	0.029*	
C229	0.8729 (4)	0.77340 (13)	0.94048 (18)	0.0210 (8)	
H229	0.883	0.8054	0.9279	0.025*	
C230	0.7504 (4)	0.82926 (13)	0.81709 (18)	0.0191 (8)	
C231	0.7240 (4)	0.86965 (13)	0.85781 (18)	0.0192 (8)	
C232	0.6607 (4)	0.86690 (14)	0.91772 (19)	0.0230 (8)	
H232	0.6353	0.8369	0.9329	0.028*	
C233	0.6359 (4)	0.90717 (14)	0.9537 (2)	0.0266 (9)	
H233	0.593	0.9042	0.9925	0.032*	
C234	0.6745 (4)	0.95301 (15)	0.9329 (2)	0.0323 (10)	
H234	0.6566	0.9803	0.9575	0.039*	
C235	0.7385 (4)	0.95739 (14)	0.8763 (2)	0.0300 (10)	
H235	0.7655	0.9878	0.8633	0.036*	
C236	0.7646 (4)	0.91686 (13)	0.8371 (2)	0.0240 (8)	
C237	0.8269 (4)	0.92113 (15)	0.7772 (2)	0.0311 (10)	
H237	0.8497	0.9516	0.7625	0.037*	
C238	0.8541 (4)	0.88163 (15)	0.74067 (19)	0.0272 (9)	
H238	0.8979	0.8852	0.7022	0.033*	
C239	0.8161 (4)	0.83557 (14)	0.76091 (19)	0.0236 (8)	
H239	0.8359	0.8088	0.7358	0.028*	
O1	0.4643 (3)	0.70580 (10)	0.69001 (15)	0.0304 (7)	
O2	0.2950 (4)	0.97818 (12)	0.93165 (17)	0.0539 (10)	
O3	0.3165 (4)	0.57932 (12)	0.86447 (18)	0.0503 (9)	
C43	−0.0219 (6)	0.97133 (19)	0.5102 (2)	0.0549 (15)	
H43A	−0.0012	0.9434	0.4849	0.082*	
H43B	0.0383	0.997	0.5018	0.082*	
H43C	−0.111	0.9814	0.4974	0.082*	
P1	0.81469 (9)	0.65586 (3)	0.67416 (5)	0.01594 (19)	
P2	0.69062 (9)	0.76754 (3)	0.82986 (5)	0.01584 (19)	
Cl1	0.96409 (10)	0.72128 (4)	0.78862 (5)	0.0230 (2)	
Rh1	0.74079 (2)	0.712399 (9)	0.748699 (15)	0.01574 (6)	

### Atomic displacement parameters (Å^2^)

**Table:**

	*U*^11^	*U*^22^	*U*^33^	*U*^12^	*U*^13^	*U*^23^
C1	0.025 (2)	0.0116 (18)	0.0235 (19)	0.0012 (15)	0.0063 (17)	−0.0043 (15)
C10	0.021 (2)	0.0191 (19)	0.0158 (18)	−0.0001 (16)	−0.0021 (15)	0.0008 (15)
C21	0.066 (4)	0.036 (3)	0.072 (4)	0.010 (3)	−0.014 (3)	−0.022 (3)
C22	0.045 (3)	0.025 (2)	0.034 (2)	−0.016 (2)	−0.003 (2)	−0.0010 (18)
C23	0.034 (3)	0.065 (4)	0.088 (5)	0.001 (3)	−0.008 (3)	−0.012 (3)
C31	0.043 (3)	0.050 (3)	0.028 (2)	−0.014 (2)	0.005 (2)	−0.010 (2)
C32	0.043 (3)	0.030 (2)	0.038 (2)	0.008 (2)	0.013 (2)	0.0009 (19)
C33	0.025 (2)	0.032 (2)	0.035 (2)	−0.0050 (19)	−0.0013 (19)	−0.0030 (19)
C41	0.114 (7)	0.058 (4)	0.065 (4)	0.029 (4)	−0.009 (4)	−0.014 (3)
C42	0.090 (5)	0.020 (2)	0.049 (3)	0.005 (3)	0.029 (3)	−0.002 (2)
O4	0.147 (5)	0.035 (2)	0.096 (3)	−0.006 (3)	0.071 (3)	0.006 (2)
C110	0.0137 (18)	0.0176 (18)	0.0234 (18)	0.0017 (14)	−0.0009 (15)	−0.0011 (15)
C111	0.0167 (18)	0.0091 (16)	0.0266 (19)	0.0036 (14)	−0.0013 (15)	−0.0014 (14)
C112	0.024 (2)	0.0182 (19)	0.023 (2)	−0.0004 (16)	0.0022 (17)	−0.0019 (16)
C113	0.026 (2)	0.0181 (19)	0.030 (2)	−0.0016 (16)	0.0035 (17)	−0.0002 (16)
C114	0.020 (2)	0.0164 (19)	0.038 (2)	−0.0047 (16)	0.0043 (17)	0.0019 (17)
C115	0.019 (2)	0.0167 (19)	0.038 (2)	−0.0020 (15)	−0.0074 (17)	−0.0010 (17)
C116	0.020 (2)	0.0140 (17)	0.028 (2)	−0.0014 (15)	−0.0047 (16)	−0.0013 (15)
C117	0.023 (2)	0.0172 (19)	0.027 (2)	−0.0009 (16)	−0.0126 (16)	−0.0029 (16)
C118	0.025 (2)	0.020 (2)	0.0199 (19)	−0.0008 (17)	−0.0060 (16)	0.0028 (16)
C119	0.0201 (19)	0.0168 (18)	0.0204 (18)	−0.0006 (15)	−0.0024 (15)	0.0016 (15)
C120	0.0178 (19)	0.0179 (18)	0.0199 (18)	−0.0030 (15)	−0.0013 (15)	0.0029 (15)
C121	0.0171 (19)	0.0182 (19)	0.0262 (19)	−0.0038 (15)	−0.0035 (15)	0.0021 (15)
C122	0.025 (2)	0.023 (2)	0.0239 (19)	0.0032 (17)	−0.0004 (16)	−0.0006 (16)
C123	0.034 (3)	0.025 (2)	0.032 (2)	0.0002 (18)	−0.0005 (19)	−0.0054 (17)
C124	0.033 (3)	0.022 (2)	0.053 (3)	0.0102 (19)	−0.006 (2)	−0.005 (2)
C125	0.036 (3)	0.022 (2)	0.043 (3)	0.0028 (19)	−0.010 (2)	0.0048 (19)
C126	0.022 (2)	0.021 (2)	0.030 (2)	−0.0021 (16)	−0.0054 (17)	0.0023 (16)
C127	0.029 (2)	0.022 (2)	0.038 (2)	−0.0035 (18)	−0.0058 (19)	0.0123 (18)
C128	0.028 (2)	0.030 (2)	0.025 (2)	0.0008 (18)	−0.0033 (17)	0.0029 (17)
C129	0.022 (2)	0.0195 (19)	0.021 (2)	−0.0006 (16)	−0.0005 (16)	0.0015 (16)
C130	0.0160 (18)	0.0176 (18)	0.0179 (17)	−0.0054 (14)	0.0000 (14)	−0.0004 (14)
C131	0.022 (2)	0.0123 (16)	0.0151 (16)	−0.0047 (15)	−0.0031 (14)	−0.0024 (14)
C132	0.020 (2)	0.0214 (19)	0.0156 (17)	−0.0045 (16)	−0.0014 (15)	−0.0012 (15)
C133	0.022 (2)	0.0194 (19)	0.024 (2)	0.0002 (16)	−0.0034 (16)	−0.0022 (16)
C134	0.032 (2)	0.0180 (19)	0.0214 (19)	−0.0020 (17)	−0.0032 (17)	0.0009 (15)
C135	0.026 (2)	0.0226 (19)	0.0200 (18)	−0.0088 (16)	0.0008 (16)	−0.0007 (15)
C136	0.023 (2)	0.021 (2)	0.0153 (18)	−0.0053 (16)	−0.0001 (16)	−0.0064 (15)
C137	0.021 (2)	0.027 (2)	0.0223 (19)	−0.0079 (17)	0.0047 (16)	−0.0023 (16)
C138	0.020 (2)	0.023 (2)	0.027 (2)	0.0020 (16)	0.0017 (16)	−0.0060 (16)
C139	0.020 (2)	0.0191 (18)	0.0210 (19)	0.0017 (15)	−0.0021 (15)	−0.0064 (15)
C210	0.0165 (18)	0.0185 (18)	0.0180 (17)	−0.0011 (14)	−0.0005 (14)	0.0002 (14)
C211	0.0225 (19)	0.0129 (17)	0.0193 (17)	−0.0019 (14)	−0.0023 (15)	−0.0016 (14)
C213	0.020 (2)	0.0203 (19)	0.0232 (19)	0.0009 (15)	−0.0005 (16)	0.0005 (15)
C214	0.033 (2)	0.026 (2)	0.0179 (18)	−0.0027 (18)	−0.0045 (17)	0.0017 (16)
C215	0.034 (2)	0.026 (2)	0.0224 (19)	0.0061 (18)	−0.0145 (17)	−0.0009 (17)
C216	0.019 (2)	0.024 (2)	0.034 (2)	0.0003 (16)	−0.0084 (17)	−0.0060 (17)
C217	0.022 (2)	0.0135 (18)	0.0223 (18)	0.0013 (14)	−0.0013 (15)	−0.0048 (14)
C218	0.0185 (19)	0.022 (2)	0.028 (2)	−0.0036 (15)	−0.0006 (16)	−0.0090 (16)
C219	0.021 (2)	0.0213 (19)	0.0191 (18)	−0.0071 (16)	0.0028 (15)	−0.0022 (15)
C220	0.0144 (18)	0.0207 (18)	0.0197 (18)	0.0038 (15)	0.0010 (14)	0.0006 (15)
C221	0.022 (2)	0.0205 (18)	0.0194 (18)	0.0026 (16)	0.0064 (15)	0.0007 (15)
C222	0.029 (2)	0.021 (2)	0.0183 (19)	−0.0018 (17)	0.0045 (16)	−0.0010 (15)
C223	0.043 (3)	0.019 (2)	0.023 (2)	−0.0067 (19)	0.0062 (19)	−0.0041 (17)
C224	0.044 (3)	0.021 (2)	0.034 (2)	0.0035 (19)	0.013 (2)	0.0047 (18)
C225	0.031 (2)	0.025 (2)	0.028 (2)	0.0032 (18)	0.0067 (19)	0.0045 (18)
C226	0.022 (2)	0.0241 (19)	0.0208 (18)	0.0051 (16)	0.0055 (16)	0.0034 (15)
C227	0.018 (2)	0.035 (2)	0.026 (2)	0.0108 (17)	−0.0017 (16)	0.0066 (18)
C228	0.017 (2)	0.031 (2)	0.0246 (19)	0.0002 (18)	−0.0050 (17)	0.0008 (17)
C229	0.021 (2)	0.0189 (18)	0.0227 (19)	0.0014 (15)	0.0002 (16)	0.0017 (15)
C230	0.0181 (19)	0.0148 (17)	0.0235 (19)	−0.0066 (14)	−0.0036 (15)	0.0018 (15)
C231	0.0162 (18)	0.0153 (18)	0.0250 (19)	−0.0024 (14)	−0.0051 (15)	0.0021 (15)
C232	0.024 (2)	0.0176 (18)	0.027 (2)	0.0002 (16)	−0.0050 (16)	−0.0024 (15)
C233	0.022 (2)	0.027 (2)	0.030 (2)	0.0028 (17)	−0.0033 (17)	−0.0062 (17)
C234	0.027 (2)	0.026 (2)	0.042 (3)	0.0020 (18)	−0.007 (2)	−0.0115 (19)
C235	0.027 (2)	0.017 (2)	0.043 (3)	0.0008 (17)	−0.013 (2)	0.0022 (18)
C236	0.023 (2)	0.0159 (19)	0.031 (2)	−0.0036 (16)	−0.0065 (17)	0.0035 (16)
C237	0.027 (2)	0.025 (2)	0.039 (2)	−0.0059 (17)	−0.0077 (19)	0.0128 (19)
C238	0.028 (2)	0.027 (2)	0.026 (2)	−0.0101 (17)	−0.0012 (17)	0.0049 (17)
C239	0.020 (2)	0.024 (2)	0.027 (2)	0.0009 (16)	−0.0014 (17)	0.0075 (16)
O1	0.0229 (16)	0.0266 (16)	0.0404 (18)	−0.0012 (13)	−0.0051 (13)	−0.0105 (13)
O2	0.073 (3)	0.040 (2)	0.045 (2)	−0.0016 (19)	−0.0150 (19)	−0.0119 (17)
O3	0.060 (3)	0.0357 (19)	0.056 (2)	0.0065 (18)	0.0086 (19)	0.0126 (17)
C43	0.074 (4)	0.043 (3)	0.049 (3)	0.020 (3)	0.009 (3)	−0.006 (3)
P1	0.0155 (5)	0.0155 (5)	0.0166 (4)	−0.0012 (4)	−0.0002 (4)	0.0012 (4)
P2	0.0162 (5)	0.0146 (4)	0.0164 (4)	−0.0008 (4)	−0.0010 (4)	−0.0006 (4)
Cl1	0.0168 (5)	0.0252 (5)	0.0266 (5)	−0.0027 (4)	−0.0001 (4)	−0.0049 (4)
Rh1	0.01477 (13)	0.01523 (12)	0.01692 (11)	−0.00188 (14)	−0.00049 (9)	−0.00096 (13)

### Geometric parameters (Å, °)

**Table:**

C1—O1	1.136 (5)	C132—H132	0.93
C1—Rh1	1.823 (4)	C133—C134	1.400 (5)
C10—C210	1.384 (5)	C133—H133	0.93
C10—C219	1.391 (5)	C134—C135	1.357 (6)
C10—H10	0.93	C134—H134	0.93
C21—C22	1.488 (7)	C135—C136	1.416 (5)
C21—H21A	0.96	C135—H135	0.93
C21—H21B	0.96	C136—C137	1.411 (6)
C21—H21C	0.96	C137—C138	1.357 (5)
C22—O2	1.204 (5)	C137—H137	0.93
C22—C23	1.482 (7)	C138—C139	1.396 (5)
C23—H23A	0.96	C138—H138	0.93
C23—H23B	0.96	C139—H139	0.93
C23—H23C	0.96	C210—C211	1.428 (5)
C31—C33	1.500 (6)	C210—P2	1.827 (4)
C31—H31A	0.96	C211—C217	1.422 (5)
C31—H31B	0.96	C211—C213	1.430 (5)
C31—H31C	0.96	C213—C214	1.358 (5)
C32—C33	1.506 (6)	C213—H213	0.93
C32—H32A	0.96	C214—C215	1.410 (6)
C32—H32B	0.96	C214—H214	0.93
C32—H32C	0.96	C215—C216	1.361 (6)
C33—O3	1.202 (5)	C215—H215	0.93
C41—C42	1.479 (9)	C216—C217	1.415 (5)
C41—H41A	0.96	C216—H216	0.93
C41—H41B	0.96	C217—C218	1.417 (5)
C41—H41C	0.96	C218—C219	1.364 (5)
C42—O4	1.225 (7)	C218—H218	0.93
C42—C43	1.483 (7)	C219—H219	0.93
C110—C119	1.375 (5)	C220—C229	1.370 (5)
C110—C111	1.437 (5)	C220—C221	1.446 (5)
C110—P1	1.827 (4)	C220—P2	1.830 (4)
C111—C116	1.416 (5)	C221—C222	1.413 (6)
C111—C112	1.429 (5)	C221—C226	1.421 (5)
C112—C113	1.357 (5)	C222—C223	1.363 (5)
C112—H112	0.93	C222—H222	0.93
C113—C114	1.403 (6)	C223—C224	1.418 (6)
C113—H113	0.93	C223—H223	0.93
C114—C115	1.356 (6)	C224—C225	1.361 (6)
C114—H114	0.93	C224—H224	0.93
C115—C116	1.424 (5)	C225—C226	1.413 (6)
C115—H115	0.93	C225—H225	0.93
C116—C117	1.416 (5)	C226—C227	1.411 (6)
C117—C118	1.366 (6)	C227—C228	1.354 (6)
C117—H117	0.93	C227—H227	0.93
C118—C119	1.402 (5)	C228—C229	1.400 (5)
C118—H118	0.93	C228—H228	0.93
C119—H119	0.93	C229—H229	0.93
C120—C129	1.378 (5)	C230—C239	1.372 (5)
C120—C121	1.445 (5)	C230—C231	1.426 (5)
C120—P1	1.834 (4)	C230—P2	1.837 (4)
C121—C126	1.412 (5)	C231—C232	1.417 (5)
C121—C122	1.415 (5)	C231—C236	1.442 (5)
C122—C123	1.364 (5)	C232—C233	1.365 (5)
C122—H122	0.93	C232—H232	0.93
C123—C124	1.411 (6)	C233—C234	1.402 (6)
C123—H123	0.93	C233—H233	0.93
C124—C125	1.352 (6)	C234—C235	1.363 (6)
C124—H124	0.93	C234—H234	0.93
C125—C126	1.421 (6)	C235—C236	1.410 (6)
C125—H125	0.93	C235—H235	0.93
C126—C127	1.425 (6)	C236—C237	1.414 (6)
C127—C128	1.353 (6)	C237—C238	1.360 (6)
C127—H127	0.93	C237—H237	0.93
C128—C129	1.401 (5)	C238—C239	1.402 (5)
C128—H128	0.93	C238—H238	0.93
C129—H129	0.93	C239—H239	0.93
C130—C139	1.377 (5)	C43—H43A	0.96
C130—C131	1.423 (5)	C43—H43B	0.96
C130—P1	1.834 (4)	C43—H43C	0.96
C131—C132	1.416 (5)	P1—Rh1	2.3360 (10)
C131—C136	1.428 (5)	P2—Rh1	2.3258 (10)
C132—C133	1.372 (5)	Cl1—Rh1	2.3525 (11)
			
O1—C1—Rh1	177.9 (4)	C137—C136—C131	119.1 (3)
C210—C10—C219	121.9 (4)	C135—C136—C131	119.9 (4)
C210—C10—H10	119.1	C138—C137—C136	120.8 (4)
C219—C10—H10	119.1	C138—C137—H137	119.6
C22—C21—H21A	109.5	C136—C137—H137	119.6
C22—C21—H21B	109.5	C137—C138—C139	120.4 (4)
H21A—C21—H21B	109.5	C137—C138—H138	119.8
C22—C21—H21C	109.5	C139—C138—H138	119.8
H21A—C21—H21C	109.5	C130—C139—C138	121.6 (4)
H21B—C21—H21C	109.5	C130—C139—H139	119.2
O2—C22—C23	121.2 (5)	C138—C139—H139	119.2
O2—C22—C21	121.1 (5)	C10—C210—C211	118.8 (3)
C23—C22—C21	117.7 (5)	C10—C210—P2	119.7 (3)
C22—C23—H23A	109.5	C211—C210—P2	121.5 (3)
C22—C23—H23B	109.5	C217—C211—C210	119.0 (3)
H23A—C23—H23B	109.5	C217—C211—C213	117.5 (3)
C22—C23—H23C	109.5	C210—C211—C213	123.5 (3)
H23A—C23—H23C	109.5	C214—C213—C211	120.9 (4)
H23B—C23—H23C	109.5	C214—C213—H213	119.5
C33—C31—H31A	109.5	C211—C213—H213	119.5
C33—C31—H31B	109.5	C213—C214—C215	121.2 (4)
H31A—C31—H31B	109.5	C213—C214—H214	119.4
C33—C31—H31C	109.5	C215—C214—H214	119.4
H31A—C31—H31C	109.5	C216—C215—C214	119.5 (4)
H31B—C31—H31C	109.5	C216—C215—H215	120.3
C33—C32—H32A	109.5	C214—C215—H215	120.3
C33—C32—H32B	109.5	C215—C216—C217	121.2 (4)
H32A—C32—H32B	109.5	C215—C216—H216	119.4
C33—C32—H32C	109.5	C217—C216—H216	119.4
H32A—C32—H32C	109.5	C216—C217—C218	120.9 (4)
H32B—C32—H32C	109.5	C216—C217—C211	119.6 (3)
O3—C33—C31	121.9 (4)	C218—C217—C211	119.5 (3)
O3—C33—C32	122.4 (4)	C219—C218—C217	120.4 (4)
C31—C33—C32	115.7 (4)	C219—C218—H218	119.8
C42—C41—H41A	109.5	C217—C218—H218	119.8
C42—C41—H41B	109.5	C218—C219—C10	120.4 (4)
H41A—C41—H41B	109.5	C218—C219—H219	119.8
C42—C41—H41C	109.5	C10—C219—H219	119.8
H41A—C41—H41C	109.5	C229—C220—C221	119.0 (3)
H41B—C41—H41C	109.5	C229—C220—P2	120.4 (3)
O4—C42—C41	121.9 (6)	C221—C220—P2	119.1 (3)
O4—C42—C43	118.3 (7)	C222—C221—C226	118.4 (4)
C41—C42—C43	119.8 (5)	C222—C221—C220	123.2 (4)
C119—C110—C111	119.3 (3)	C226—C221—C220	118.3 (4)
C119—C110—P1	120.8 (3)	C223—C222—C221	121.1 (4)
C111—C110—P1	119.8 (3)	C223—C222—H222	119.4
C116—C111—C112	117.8 (3)	C221—C222—H222	119.4
C116—C111—C110	118.7 (3)	C222—C223—C224	120.6 (4)
C112—C111—C110	123.4 (4)	C222—C223—H223	119.7
C113—C112—C111	121.0 (4)	C224—C223—H223	119.7
C113—C112—H112	119.5	C225—C224—C223	119.4 (4)
C111—C112—H112	119.5	C225—C224—H224	120.3
C112—C113—C114	121.1 (4)	C223—C224—H224	120.3
C112—C113—H113	119.5	C224—C225—C226	121.5 (4)
C114—C113—H113	119.5	C224—C225—H225	119.3
C115—C114—C113	119.7 (4)	C226—C225—H225	119.3
C115—C114—H114	120.2	C227—C226—C225	121.3 (4)
C113—C114—H114	120.2	C227—C226—C221	119.7 (3)
C114—C115—C116	121.4 (4)	C225—C226—C221	119.0 (4)
C114—C115—H115	119.3	C228—C227—C226	120.8 (4)
C116—C115—H115	119.3	C228—C227—H227	119.6
C111—C116—C117	119.9 (3)	C226—C227—H227	119.6
C111—C116—C115	119.0 (4)	C227—C228—C229	120.4 (4)
C117—C116—C115	121.1 (4)	C227—C228—H228	119.8
C118—C117—C116	120.1 (4)	C229—C228—H228	119.8
C118—C117—H117	120	C220—C229—C228	121.7 (4)
C116—C117—H117	120	C220—C229—H229	119.1
C117—C118—C119	120.8 (4)	C228—C229—H229	119.1
C117—C118—H118	119.6	C239—C230—C231	120.2 (3)
C119—C118—H118	119.6	C239—C230—P2	114.9 (3)
C110—C119—C118	121.2 (4)	C231—C230—P2	124.7 (3)
C110—C119—H119	119.4	C232—C231—C230	124.7 (3)
C118—C119—H119	119.4	C232—C231—C236	117.1 (3)
C129—C120—C121	119.1 (3)	C230—C231—C236	118.2 (3)
C129—C120—P1	115.4 (3)	C233—C232—C231	121.8 (4)
C121—C120—P1	125.5 (3)	C233—C232—H232	119.1
C126—C121—C122	117.4 (3)	C231—C232—H232	119.1
C126—C121—C120	118.2 (3)	C232—C233—C234	120.7 (4)
C122—C121—C120	124.4 (3)	C232—C233—H233	119.6
C123—C122—C121	122.0 (4)	C234—C233—H233	119.6
C123—C122—H122	119	C235—C234—C233	119.7 (4)
C121—C122—H122	119	C235—C234—H234	120.2
C122—C123—C124	119.7 (4)	C233—C234—H234	120.2
C122—C123—H123	120.1	C234—C235—C236	121.5 (4)
C124—C123—H123	120.1	C234—C235—H235	119.2
C125—C124—C123	120.3 (4)	C236—C235—H235	119.2
C125—C124—H124	119.8	C235—C236—C237	122.1 (4)
C123—C124—H124	119.8	C235—C236—C231	119.2 (4)
C124—C125—C126	120.7 (4)	C237—C236—C231	118.7 (4)
C124—C125—H125	119.6	C238—C237—C236	121.4 (4)
C126—C125—H125	119.6	C238—C237—H237	119.3
C121—C126—C125	119.8 (4)	C236—C237—H237	119.3
C121—C126—C127	120.1 (4)	C237—C238—C239	120.0 (4)
C125—C126—C127	120.2 (4)	C237—C238—H238	120
C128—C127—C126	120.6 (4)	C239—C238—H238	120
C128—C127—H127	119.7	C230—C239—C238	121.3 (4)
C126—C127—H127	119.7	C230—C239—H239	119.3
C127—C128—C129	120.1 (4)	C238—C239—H239	119.3
C127—C128—H128	120	C42—C43—H43A	109.5
C129—C128—H128	120	C42—C43—H43B	109.5
C120—C129—C128	121.9 (4)	H43A—C43—H43B	109.5
C120—C129—H129	119	C42—C43—H43C	109.5
C128—C129—H129	119	H43A—C43—H43C	109.5
C139—C130—C131	119.2 (3)	H43B—C43—H43C	109.5
C139—C130—P1	119.8 (3)	C110—P1—C120	104.45 (17)
C131—C130—P1	120.4 (3)	C110—P1—C130	106.37 (17)
C132—C131—C130	123.9 (3)	C120—P1—C130	106.86 (17)
C132—C131—C136	117.1 (3)	C110—P1—Rh1	116.05 (12)
C130—C131—C136	118.9 (3)	C120—P1—Rh1	114.49 (12)
C133—C132—C131	121.4 (4)	C130—P1—Rh1	108.00 (12)
C133—C132—H132	119.3	C210—P2—C220	107.01 (16)
C131—C132—H132	119.3	C210—P2—C230	104.38 (17)
C132—C133—C134	120.8 (4)	C220—P2—C230	106.60 (17)
C132—C133—H133	119.6	C210—P2—Rh1	117.67 (12)
C134—C133—H133	119.6	C220—P2—Rh1	105.81 (12)
C135—C134—C133	119.9 (4)	C230—P2—Rh1	114.70 (12)
C135—C134—H134	120	C1—Rh1—P2	93.94 (11)
C133—C134—H134	120	C1—Rh1—P1	92.46 (11)
C134—C135—C136	120.9 (4)	P2—Rh1—P1	173.41 (4)
C134—C135—H135	119.5	C1—Rh1—Cl1	176.51 (13)
C136—C135—H135	119.5	P2—Rh1—Cl1	87.04 (3)
C137—C136—C135	121.0 (4)	P1—Rh1—Cl1	86.67 (3)
			
C119—C110—C111—C116	−1.2 (5)	P2—C220—C221—C226	166.3 (3)
P1—C110—C111—C116	175.7 (3)	C226—C221—C222—C223	−1.5 (5)
C119—C110—C111—C112	178.0 (3)	C220—C221—C222—C223	177.8 (4)
P1—C110—C111—C112	−5.1 (5)	C221—C222—C223—C224	−0.6 (6)
C116—C111—C112—C113	−1.2 (5)	C222—C223—C224—C225	2.2 (6)
C110—C111—C112—C113	179.5 (3)	C223—C224—C225—C226	−1.7 (6)
C111—C112—C113—C114	−0.1 (6)	C224—C225—C226—C227	−179.1 (4)
C112—C113—C114—C115	1.0 (6)	C224—C225—C226—C221	−0.4 (6)
C113—C114—C115—C116	−0.5 (6)	C222—C221—C226—C227	−179.3 (3)
C112—C111—C116—C117	−177.2 (3)	C220—C221—C226—C227	1.4 (5)
C110—C111—C116—C117	2.1 (5)	C222—C221—C226—C225	2.0 (5)
C112—C111—C116—C115	1.7 (5)	C220—C221—C226—C225	−177.3 (3)
C110—C111—C116—C115	−179.1 (3)	C225—C226—C227—C228	178.1 (4)
C114—C115—C116—C111	−0.9 (6)	C221—C226—C227—C228	−0.5 (5)
C114—C115—C116—C117	178.0 (4)	C226—C227—C228—C229	−1.5 (6)
C111—C116—C117—C118	−1.7 (6)	C221—C220—C229—C228	−1.8 (5)
C115—C116—C117—C118	179.5 (4)	P2—C220—C229—C228	−168.1 (3)
C116—C117—C118—C119	0.4 (6)	C227—C228—C229—C220	2.7 (6)
C111—C110—C119—C118	0.0 (5)	C239—C230—C231—C232	−177.9 (4)
P1—C110—C119—C118	−176.9 (3)	P2—C230—C231—C232	7.2 (6)
C117—C118—C119—C110	0.5 (6)	C239—C230—C231—C236	1.8 (5)
C129—C120—C121—C126	−0.8 (5)	P2—C230—C231—C236	−173.0 (3)
P1—C120—C121—C126	−179.3 (3)	C230—C231—C232—C233	−178.6 (4)
C129—C120—C121—C122	180.0 (4)	C236—C231—C232—C233	1.6 (6)
P1—C120—C121—C122	1.5 (6)	C231—C232—C233—C234	−0.9 (6)
C126—C121—C122—C123	−0.9 (6)	C232—C233—C234—C235	−0.6 (6)
C120—C121—C122—C123	178.4 (4)	C233—C234—C235—C236	1.4 (6)
C121—C122—C123—C124	−0.5 (6)	C234—C235—C236—C237	177.8 (4)
C122—C123—C124—C125	2.2 (7)	C234—C235—C236—C231	−0.7 (6)
C123—C124—C125—C126	−2.4 (7)	C232—C231—C236—C235	−0.8 (6)
C122—C121—C126—C125	0.7 (6)	C230—C231—C236—C235	179.4 (4)
C120—C121—C126—C125	−178.6 (4)	C232—C231—C236—C237	−179.3 (4)
C122—C121—C126—C127	−179.4 (4)	C230—C231—C236—C237	0.9 (6)
C120—C121—C126—C127	1.3 (6)	C235—C236—C237—C238	178.5 (4)
C124—C125—C126—C121	1.0 (7)	C231—C236—C237—C238	−2.9 (6)
C124—C125—C126—C127	−179.0 (4)	C236—C237—C238—C239	2.3 (7)
C121—C126—C127—C128	−0.4 (6)	C231—C230—C239—C238	−2.6 (6)
C125—C126—C127—C128	179.6 (4)	P2—C230—C239—C238	172.7 (3)
C126—C127—C128—C129	−1.1 (6)	C237—C238—C239—C230	0.5 (6)
C121—C120—C129—C128	−0.7 (6)	C119—C110—P1—C120	−120.0 (3)
P1—C120—C129—C128	178.0 (3)	C111—C110—P1—C120	63.2 (3)
C127—C128—C129—C120	1.7 (6)	C119—C110—P1—C130	−7.1 (3)
C139—C130—C131—C132	−178.4 (3)	C111—C110—P1—C130	176.0 (3)
P1—C130—C131—C132	−7.7 (5)	C119—C110—P1—Rh1	113.0 (3)
C139—C130—C131—C136	−1.3 (5)	C111—C110—P1—Rh1	−63.9 (3)
P1—C130—C131—C136	169.4 (3)	C129—C120—P1—C110	−132.0 (3)
C130—C131—C132—C133	176.5 (3)	C121—C120—P1—C110	46.5 (4)
C136—C131—C132—C133	−0.6 (5)	C129—C120—P1—C130	115.6 (3)
C131—C132—C133—C134	0.6 (6)	C121—C120—P1—C130	−65.9 (4)
C132—C133—C134—C135	0.3 (6)	C129—C120—P1—Rh1	−4.0 (3)
C133—C134—C135—C136	−1.1 (6)	C121—C120—P1—Rh1	174.6 (3)
C134—C135—C136—C137	−178.5 (4)	C139—C130—P1—C110	−110.0 (3)
C134—C135—C136—C131	1.0 (6)	C131—C130—P1—C110	79.4 (3)
C132—C131—C136—C137	179.4 (3)	C139—C130—P1—C120	1.1 (3)
C130—C131—C136—C137	2.1 (5)	C131—C130—P1—C120	−169.5 (3)
C132—C131—C136—C135	−0.1 (5)	C139—C130—P1—Rh1	124.8 (3)
C130—C131—C136—C135	−177.4 (3)	C131—C130—P1—Rh1	−45.8 (3)
C135—C136—C137—C138	178.4 (4)	C10—C210—P2—C220	−7.8 (3)
C131—C136—C137—C138	−1.1 (6)	C211—C210—P2—C220	174.4 (3)
C136—C137—C138—C139	−0.7 (6)	C10—C210—P2—C230	−120.5 (3)
C131—C130—C139—C138	−0.5 (5)	C211—C210—P2—C230	61.7 (3)
P1—C130—C139—C138	−171.2 (3)	C10—C210—P2—Rh1	111.0 (3)
C137—C138—C139—C130	1.5 (6)	C211—C210—P2—Rh1	−66.7 (3)
C219—C10—C210—C211	1.8 (6)	C229—C220—P2—C210	−116.3 (3)
C219—C10—C210—P2	−176.0 (3)	C221—C220—P2—C210	77.5 (3)
C10—C210—C211—C217	−3.9 (5)	C229—C220—P2—C230	−5.0 (3)
P2—C210—C211—C217	173.8 (3)	C221—C220—P2—C230	−171.3 (3)
C10—C210—C211—C213	175.5 (3)	C229—C220—P2—Rh1	117.5 (3)
P2—C210—C211—C213	−6.7 (5)	C221—C220—P2—Rh1	−48.8 (3)
C217—C211—C213—C214	0.1 (5)	C239—C230—P2—C210	−129.5 (3)
C210—C211—C213—C214	−179.4 (4)	C231—C230—P2—C210	45.5 (4)
C211—C213—C214—C215	−1.6 (6)	C239—C230—P2—C220	117.4 (3)
C213—C214—C215—C216	1.1 (6)	C231—C230—P2—C220	−67.5 (4)
C214—C215—C216—C217	1.0 (6)	C239—C230—P2—Rh1	0.7 (3)
C215—C216—C217—C218	176.4 (4)	C231—C230—P2—Rh1	175.7 (3)
C215—C216—C217—C211	−2.6 (5)	C210—P2—Rh1—C1	14.29 (18)
C210—C211—C217—C216	−178.5 (3)	C220—P2—Rh1—C1	133.75 (17)
C213—C211—C217—C216	2.0 (5)	C230—P2—Rh1—C1	−109.06 (18)
C210—C211—C217—C218	2.5 (5)	C210—P2—Rh1—Cl1	−169.07 (14)
C213—C211—C217—C218	−177.0 (3)	C220—P2—Rh1—Cl1	−49.61 (13)
C216—C217—C218—C219	−177.8 (4)	C230—P2—Rh1—Cl1	67.58 (14)
C211—C217—C218—C219	1.1 (5)	C110—P1—Rh1—C1	5.02 (18)
C217—C218—C219—C10	−3.4 (6)	C120—P1—Rh1—C1	−116.85 (18)
C210—C10—C219—C218	1.9 (6)	C130—P1—Rh1—C1	124.26 (18)
C229—C220—C221—C222	−179.5 (3)	C110—P1—Rh1—Cl1	−171.60 (13)
P2—C220—C221—C222	−13.0 (5)	C120—P1—Rh1—Cl1	66.54 (13)
C229—C220—C221—C226	−0.2 (5)	C130—P1—Rh1—Cl1	−52.35 (13)
